# The nucleoprotein of influenza A virus induces p53 signaling and apoptosis via attenuation of host ubiquitin ligase RNF43

**DOI:** 10.1038/cddis.2015.131

**Published:** 2015-05-21

**Authors:** H Nailwal, S Sharma, A K Mayank, S K Lal

**Affiliations:** 1 School of Science, Monash University Malaysia, Bandar Sunway, 47500 Petaling Jaya, Selangor DE, Malaysia; 2 Virology Group, International Centre for Genetic Engineering and Biotechnology, Aruna Asaf Ali Marg, New Delhi 110067, India

## Abstract

The interplay between influenza virus and host factors to support the viral life cycle is well documented. Influenza A virus (IAV) proteins interact with an array of cellular proteins and hijack host pathways which are at the helm of cellular responses to facilitate virus invasion. The multifaceted nature of the ubiquitination pathway for protein regulation makes it a vulnerable target of many viruses including IAV. To this end we conducted a yeast two-hybrid screen to search for cellular ubiquitin ligases important for influenza virus replication. We identified host protein, RING finger protein 43 (RNF43), a RING-type E3 ubiquitin ligase, as a novel interactor of nucleoprotein (NP) of IAV and an essential partner to induce NP-driven p53-mediated apoptosis in IAV-infected cells. In this study, we demonstrate that IAV leads to attenuation of RNF43 transcripts and hence its respective protein levels in the cellular milieu whereas in RNF43 depleted cells, viral replication was escalated several folds. Moreover, RNF43 polyubiquitinates p53 which further leads to its destabilization resulting in a decrease in induction of the p53 apoptotic pathway, a hitherto unknown process targeted by NP for p53 stabilization and accumulation. Collectively, these results conclude that NP targets RNF43 to modulate p53 ubiquitination levels and hence causes p53 stabilization which is conducive to an enhanced apoptosis level in the host cells. In conclusion, our study unravels a novel strategy adopted by IAV for utilizing the much conserved ubiquitin proteasomal pathway.

Influenza A virus (IAV) by far remains the most important of all respiratory viruses, causing severe morbidity and mortality every year.^1^ Nucleoprotein (NP) of IAV is a viral RNA genome-encapsulating structural protein that has been implicated in various other indispensable activities for virus replication and pathogenesis-like intracellular trafficking of the viral genome, viral RNA replication, virus assembly^2, 3^ via its interaction with a plethora of cellular factors like cytoskeleton scaffolding protein α-actinin-4, nuclear import receptor α importin, nuclear export receptor CRM1, DEAD-box helicase BAT1/UAP56 and cytoskeletal element F actin.^4, 5, 6, 7, 8^

The evolution of host–microbe interaction is mediated through the orchestration of different viral and host signaling pathways. Similarly, IAV requires an intricate regulatory network of viral and cellular proteins to accomplish successful replication.^9^ One of the pathways that have been shown to be maneuvered by the virus is the ubiquitin (Ub) proteasomal pathway (UPP). UPP is a multi-enzyme cascade that involves the sequential action of three different enzymes: E1 ubiquitin-activating enzyme, E2 ubiquitin-conjugating enzyme and E3 ubiquitin ligase. A completely different class of proteins, known as deubiquitinases, reverses this process by removing the Ub molecules from target proteins.^10^ With 400 putative E3 ligases encoded by the mammalian genome, the receptor specificity is maintained by these proteins and hence, are heavily exploited by viruses.^11^ IAV protein NS1 is reported to target ubiquitin ligase, TRIM 25 to escape RIG1 recognition^12^ and M1 protein reportedly interacts with E3 ligase, Itch.^13^ Moreover, recently it has been discovered that NP stabilizes the tumor suppressor protein, p53 through its decreased ubiquitination by ubiquitin ligase, MDM2.^14^ In this study, we have identified that NP interacts with E3 ubiquitin ligase, RNF43. RNF43 is a recently identified member of the RING finger family of ubiquitin ligases and has been implicated to be overexpressed in human colorectal and hepatocellular carcinomas with anti-apoptotic and growth-promoting effects that also interacts with HAP95 and NEDL1, an upstream effector of p53.^15, 16, 17, 18, 19^ A crucial mediator of cellular apoptosis, p53 is present in a latent form in unstressed cells and is regulated through various post-translational modifications like phosphorylation, ubiquitination, sumolyation, neddylation, methylation, acetylation and glycosylation of p53 polypeptide.^20, 21, 22, 23^ Although accumulation of p53 in IAV-infected cells has been demonstrated,^24, 25, 26^ it is still not clear whether IAV-induced accumulation of p53 is correlated with its activation and consecutive transactivation of its target genes that could in turn induce apoptosis in infected cells that is considered to be a peculiar feature of IAV pathogenesis.^27, 28, 29, 30, 31^

Our study provides evidences that RNF43 prevents cell death by ubiquitinating p53 and thus destabilizing it, *in-vitro*. Importantly, we have shown that IAV attenuates RNF43 on interacting with NP thus stabilizing p53 protein and consequently induces p53 signaling and apoptosis in the host cells. Identification of this new NP–RNF43 interaction could become a promising target for antiviral intervention.

## Results

### Nucleoprotein of Influenza A virus interacts with human ubiquitin ligase protein RNF43 in a conserved manner

**Figure 1:**
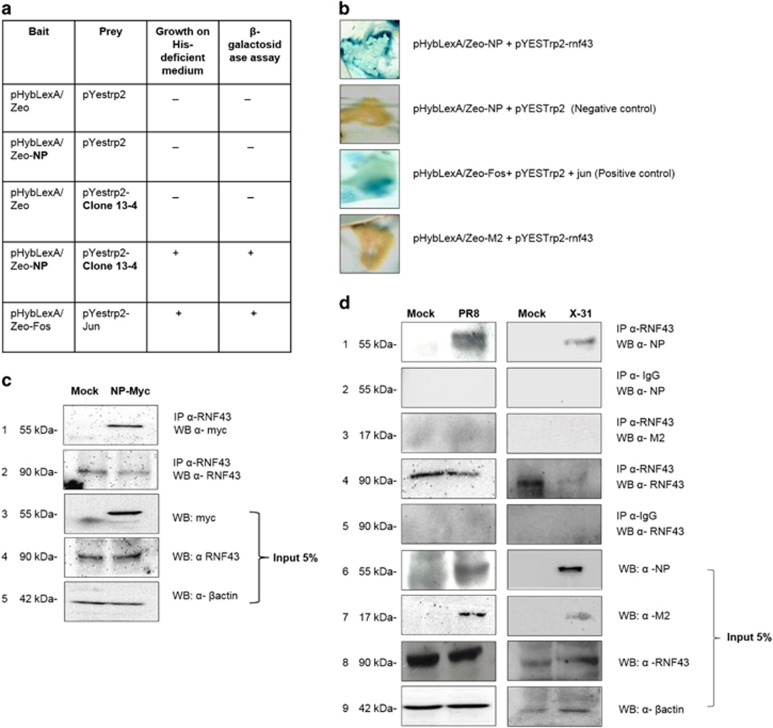
IAV NP interacts with RNF43. (**a**) Tabular representation of the yeast two-hybrid screening of lung cDNA library using NP as the bait protein. The filter *β*-gal assay for the clone 13-4 is shown as the last column of the table. (**b**) pHybLexA/Zeo-NP and pYESTrp2-RNF43 were cotransformed in L-40 yeast strain and their interaction was verified by *β*-galactosidase assay. (**c**) Lung epithelial A549 cells were transfected with either empty vector pCDNA3.1 (Mock) or pCDNA3.1-Myc-NP (NP-Myc). Forty-eight hours post transfection, cells were lysed and the lysates were subjected to co-immunoprecipitation with anti-RNF43 antibody followed by Western blotting with anti-Myc antibody. (**d**) A549 cells were infected with PR8 virus and X-31 virus at 1 MOI for 24 h and the whole-cell lysates were used for co-immunoprecipitation assays. Lysates (5%) from the same experiment were subjected to Western blotting with anti-NP (panel 6), anti-M2 (panel 7), anti-RNF43 (panel 8) and anti-*β*actin (panel 9) antibodies to show the cellular levels of these proteins

Yeast two-hybrid screening of a human cDNA lung library was conducted to identify potential interacting partners of NP of IAV. We screened a human lung cDNA library using NP from highly pathogenic A/Chicken/Hatay/2004 (H5N1), cloned into plasmid pHybLexA/Zeo, as the bait (Figure 1a). A clone encoding 1071–1622 amino acid region of RNF43 was obtained as the positive interactor which was further confirmed by BLAST analysis.

Full-length RNF43 cDNA was cloned in-frame with the activation domain pYESTrp2 and both NP–pHLZ and RNF43–pYESTrp2 were cotransformed in L-40 strain of *Saccharomyces cerevisiae*. It tested positive for histidine prototrophy and *β*-galactosidase activity confirming the interaction of full-length RNF43 with NP (Figure 1b).

The NP–RNF43 interaction was further validated by performing co-immunoprecipitation assays. HEK293T cells were transfected with pCDNA3.1-Myc-NP plasmid construct. Myc-tagged NP expression was confirmed using Western blotting as shown in Figure 1c (panel 3). Immunoprecipitation using anti-RNF43 antibody followed by immunoblotting with anti-myc antibody confirmed the interaction of RNF43 with NP (panel 1).

To validate the existence of the said interaction during IAV infection, mammalian lung epithelial A549 cells were infected with A/Puerto Rico/8/34 virus (H1N1; PR8) and A/Aichi/2/1968 virus (H3N2; X-31) at 1 MOI. The cell lysates were harvested 24 h post infection (24 h.p.i) and subjected to co-immunoprecipitation with anti-RNF43 antibody. NP protein immunoprecipitated with RNF43 antibody thus confirming that NP–RNF43 interaction was conserved between both the virus isolates (Figure 1d, panel 1). Also, co-immunoprecipitation with another IAV protein, M2 (Figure 1d, panel 3), showed that RNF43 antibody was unable to bind the M2 protein in the IAV-infected lysate, proving that RNF43 interacts exclusively with NP of IAV.

### RNF43 co-localizes with NP in the nucleus

**Figure 2:**
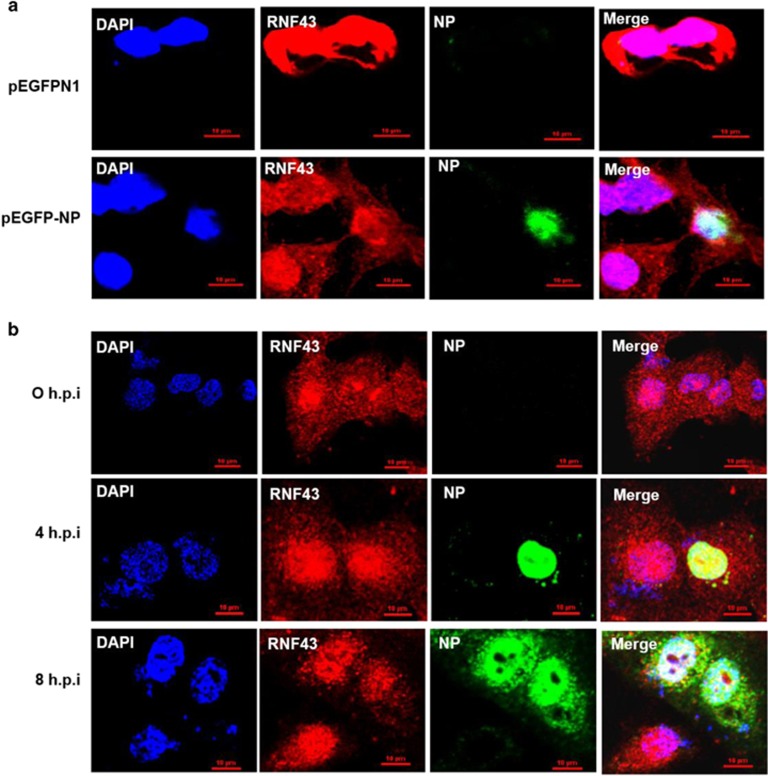
IAV NP and RNF43 co-localize in the nucleus of mammalian cells. (**a**) A549 cells were transfected with pEGFPN1 control plasmid or pEGFP-NP. Cells were fixed after 48 h and stained with DAPI for nucleus and anti-goat secondary antibody conjugated to Alexa-594 for RNF43 (red) and observed under confocal microscope. GFP-tagged NP is shown in green. (**b**) A549 cells were infected with PR8 IAV with 5 MOI and were fixed at the indicated time points. NP was stained using anti-NP monoclonal primary antibody and anti-mouse Alexa488 conjugated secondary antibody (green). RNF43 was stained using specific primary antibody and anti-rabbit Alexa-594 conjugated secondary antibody (red). Panels are labeled for their respective staining, RNF43 (red), NP (green) and nucleus (blue)

We sought to ascertain the kinetics and site of interaction of NP and RNF43 after confirming the interaction of these two proteins. A549 cells were transfected with control plasmid pEGFPN1 or pEGFP-NP followed by fixing cells 48 h post transfection and further processing for immunofluorescence analysis. RNF43 was seen to have ubiquitous nucleocytoplasmic localization in mock transfected cells (Figure 2a). However, GFP-tagged NP localized in the nucleus and co-localized with RNF43 majorly inside the nucleus.

Similarly, A549 cells were infected with PR8 at an MOI of 5 and were fixed at 0, 4 and 8 h.p.i (Figure 2b). At 4 h.p.i, NP has been observed to localize within the nucleus. At later stages of infection it is reported to move into the cytoplasm (8 h.p.i).^3^ RNF43 as previously observed had nucleocytoplasmic localization but was noted to concentrate inside the nucleus on virus infection (4 h.p.i; Figure 2b). At 8 h.p.i, the two proteins were seen to co-localize inside the nucleus. Thus, it is inferred that the primary site of interaction for the IAV NP and RNF43 is the nucleus of the host cell.

### Influenza infection decreases RNF43 mRNA and protein expression

**Figure 3:**
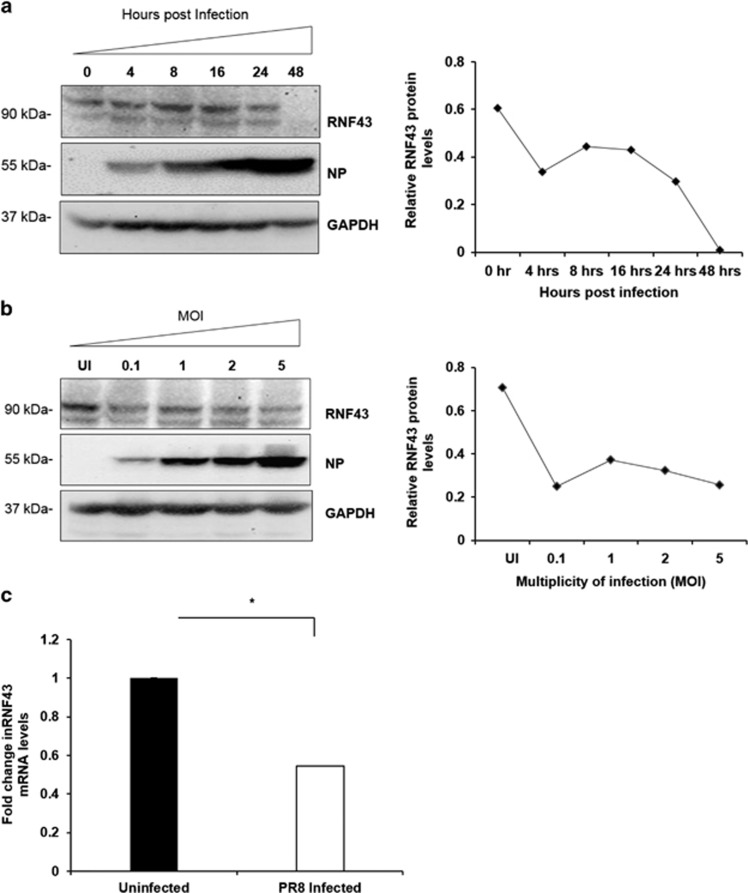
IAV infection decreases abundance of RNF43 at both mRNA and protein levels. (**a**) Lung epithelial A549 cells were infected with PR8 virus at an MOI of 1 and cells were harvested at indicated time intervals post infection and the whole-cell lysate was resolved on SDS-PAGE for Western blot analysis of RNF43, NP and GAPDH. (**b**) A549 cells were infected with PR8 virus at indicated MOIs and harvested at 24-h post infection for Western blot analysis of RNF43, NP and GAPDH. Quantitative representation of the immunoblots of both the experiments is shown as the line diagram after normalization with GAPDH (extreme right). (**c**) A549 cells were mock infected or infected with PR8 virus for 24 h and total RNA was extracted followed by rnf43 mRNA estimation with qRT-PCR. Results are shown as mean of ±S.D. of three independent experiments. * indicates statistically significant difference at *P*<0.05

To determine the physiological relevance of this interaction in the influenza virus life cycle, Western blot analysis of the PR8-infected A549 cells harvested at different time points post infection was done (Figure 3a). It showed a gradual decline in RNF43 protein expression with progression of infection reaching to its lowest levels at 48 h.p.i in the infected cells. Using the same technique, protein expression of RNF43 at different MOI of virus was evaluated (Figure 3b). A significant decrease in RNF43 protein levels with increasing virus MOI was in agreement with our previous observation. Protein levels were quantified using densitometric analysis on Image J software (1.46r, NIH, Bethesda, MD, USA).

Subsequently, we studied the mRNA levels of RNF43 after IAV infection. Total mRNA of PR8-infected A549 cells was extracted 24 h.p.i and quantified with quantitative real-time reverse transcription PCR (qRT-PCR). A remarkable 0.5-fold decrease in RNF43 mRNA levels was clearly observed as compared with the mock (Figure 3c).

These contemporaneous regulations of RNF43 transcripts and protein expression levels are indicative of an antagonistic interaction between NP and RNF43 where RNF43 is being attenuated with the progression of virus pathogenesis.

### RNF43 acts as an antiviral protein to subside viral replication

**Figure 4:**
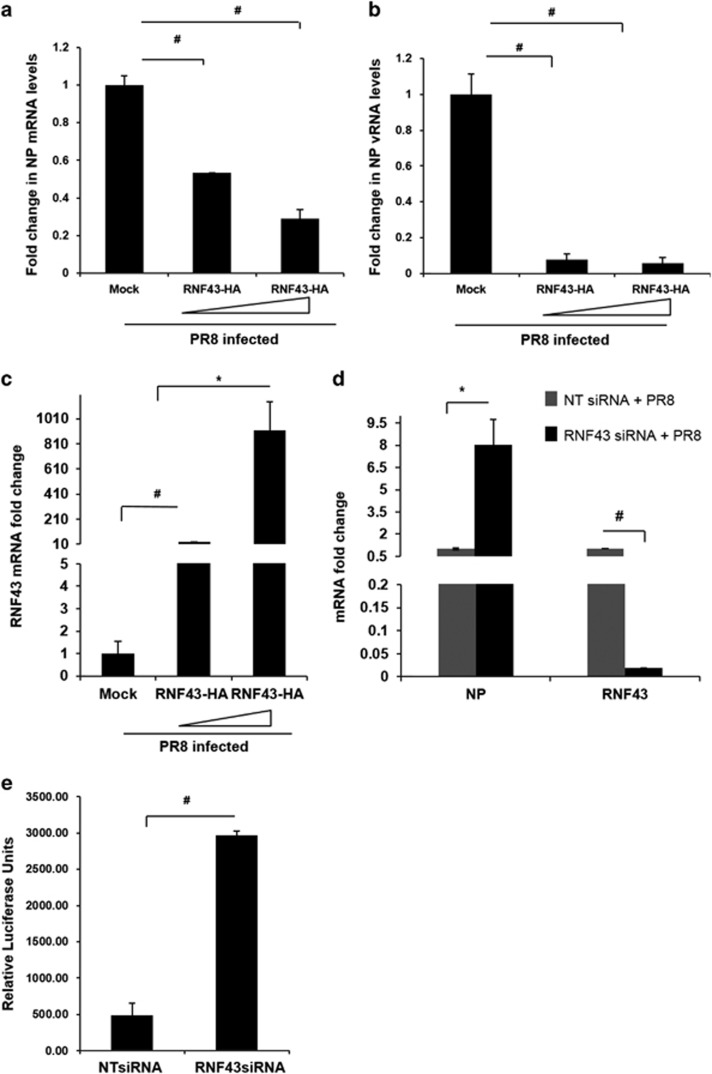
Host factor RNF43 decreases IAV replication. (**a–c**) HEK293T cells were transiently transfected with plasmid pCDNA3.1 (Mock) or pCDNA3.1-RNF43-Flag-HA (RNF43-HA) (1 and 2 *μ*g) followed by PR8 infection and incubated for 24 h. Total RNA was isolated and NP mRNA (**a**), NP vRNA (**b**) and RNF43 mRNA(**c**) levels were estimated through qRT PCR. (**d**) HEK293T cells were treated with NT siRNA or RNF43 siRNA for 24 h followed by PR8 IAV infection for next 24 h. Cells were processed for qRT-PCR analysis of mRNA levels of NP and RNF43. (**e**) A549 cells, pretreated with either NT or RNF43 siRNA were transfected with plasmids-encoding polymerase complex components (PA, PB1, PB2 and NP) derived from PR8 (H1N1 virus) were co-transfected alongside a reporter plasmid containing noncoding sequence from the NS1 segment of influenza A virus and luciferase gene driven by the Pol 1 promoter. The relative luciferase units were calculated after normalization with plasmid pRL-TK (Promega, Madison, WI, USA), which expresses Renilla luciferase that was transfected along. Results are shown as mean±S.D. of three independent experiments. * and ^#^ indicate statistically significant difference at *P*<0.05 and *P*<0.01, respectively

Viruses manipulate host proteins to their advantage often by increasing their efficiency of replication thus leading to higher virus titer in infected cells.^4^ In order to investigate this aspect, we monitored viral replication after transiently transfecting with pCDNA3.1-RNF43-Flag-HA plasmid at indicated increasing concentrations in HEK293T cells (Figures 4a–c). Empty vector pCDNA3.1 was used as a negative control. After 24 h of incubation, cells were infected with PR8 virus and harvested at 24 h. Quantification of mRNA and vRNA levels of NP was performed through real-time PCR after RNA isolation. RNF43 overexpression resulted in a gradient and a considerable decrease in NP mRNA and vRNA levels with 0.3- and 0.06-fold being the maximum recorded, respectively (Figures 4a and b). The RNF43 overexpression was confirmed with RT-PCR analysis of RNF43 mRNA levels (Figure 4c).

Next, we observed a ~8-fold increase in NP mRNA levels with ~98% silencing of RNF43 (Figure 4d). This drastic alteration in NP mRNA as well as vRNA levels exhibits a convincing antiviral role for RNF43.

In the view of above results we decided to check the effect of RNF43 on influenza virus replicase activity. A549 cells were treated with either non-targeting siRNA (NT siRNA) or RNF43 siRNA followed by co-transfection with plasmids encoding the PR8 polymerase complex genes PB2, PB1, PA and NP in conjunction with a reporter plasmid containing untranslated region of NS1 segment upstream of the luciferase gene driven by the human RNA pol I promoter. Figure 4e shows that there was a sixfold increase in replicase activity in RNF43 siRNA-treated cells as compared with the NT siRNA-treated cells. These findings strongly suggest that RNF43 is a strong inhibitor of virus replication and has antiviral properties.

### NP causes p53 stabilization by decreasing p53 ubiquitination via RNF43 ubiquitin ligase

**Figure 5:**
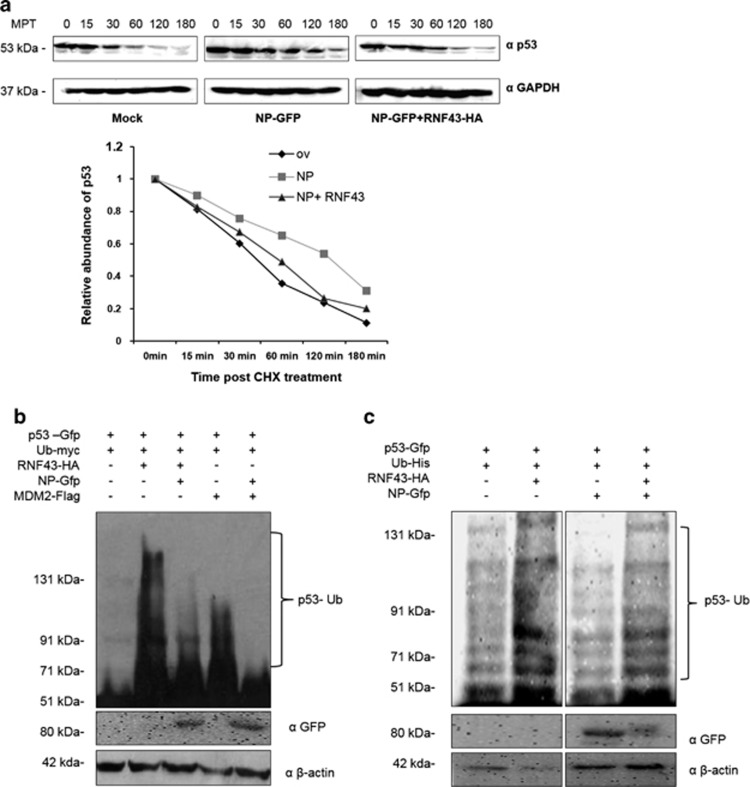
NP interacts with RNF43 to stabilize p53 through compromised ubiquitination of p53 by RNF43. (**a**) A549 cells were transiently transfected with plasmids pEGFPN1 (Mock), pEGFP-NP (NP-GFP) or pCDNA3.1-RNF43-Flag-HA (RNF43-HA) for 48 h. The transfectants were treated with cycloheximide at 50 *μ*g/ml for the indicated times (minutes) post treatment (mpt) and subjected to Western blot analysis using the indicated antibodies. The quantitative data of immunoblots is shown as line diagram after normalization with GAPDH expression levels. (**b**) HEK293T cells were transiently transfected with a combination of indicated plasmids and incubated for 48 h. The transfectants were treated with 20 *μ*g/ml MG132, 5 h before harvest. The cell lysates were subjected to Western blot analysis using the indicated antibodies. (**c**) HEK293T cells were transiently transfected with a combination of indicated plasmids and incubated for 48 h. The transfectants were treated with 20 *μ*g/ml MG132, 5 h before harvest. Ubiquitinated p53 (Ub-p53) was pulled down using Ni^2+^–NTA–agarose beads and analyzed by Western blotting with indicated antibodies

The anti-apoptotic characteristics of RNF43 protein gave us insights to ascertain the importance of the NP–RNF43 interaction in p53 stabilization and signaling. To assess p53 protein stability, 50 *μ*g/ml cycloheximide, an inhibitor of protein synthesis in eukaryotic cells, was administered to A549 cells which were transiently transfected with different expression plasmids, pEGFPN1, pEGFP-NP with or without pCDNA3.1-RNF43-Flag-HA. The half-life of p53 protein was deduced by monitoring its decay over a period of 3 h by Western blot analysis. Half-life of p53 in the NP-GFP and RNF43-Flag-HA co-transfected cells was reduced to 60 min as compared with 120 min in the case of only NP-GFP-transfected cells (Figure 5a). This data clearly proves that p53 is getting stabilized in the NP-transfected cells through the latter's interaction with RNF43 suggesting that NP–RNF43 interaction primarily leads to an enhanced p53 stabilization and accumulation.

Post-translational regulation of p53 via UPP mediated by different E3 ligases is a well-established phenomenon.^22, 23^ Because of RNF43 protein's E3 ligase activity and a RING domain in its structure we were keen to look at its role in post-translational modification of p53 through ubiquitination and explore the possibility of p53 being a substrate of its ligase activity. To this end, we first checked p53 transcript levels in A549 cells where RNF43-HA was transiently expressed. Quantitative RT-PCR analysis confirmed that RNF43 was unable to cause any significant alteration in p53 transcription (Supplementary Figure S1).

We next examined p53 ubiquitination status. HEK293T cells were treated with 20 *μ*M MG132, proteasomal inhibitor, after co-transfection with plasmids expressing p53-GFP, Ub-myc, RNF43-HA, NP-GFP and MDM2-Flag in different combinations (Figure 5b) followed by Western blot analysis of harvested cells. We observed a significant increase in ubiquitination status of p53 under RNF43 overexpression. However, NP-alleviated RNF43 caused p53 ubiquitination when transfected along with RNF43. MDM2 served as a positive control.^32^

Next, we examined the ubiquitination status of p53 through Ni–NTA pull down-based ubiquitination assay. HEK293T cells were co-transfected with a combination of plasmids expressing p53-GFP, RNF43-HA, NP-GFP along with Ub-His-expressing plasmid. Cells were harvested at 48 h.p.i after treatment with MG132, 5 h before harvesting. Ubiquitinated p53 was pulled with Ni–NTA beads and analyzed through Western blotting. As shown in Figure 5c, ladder of poly-ubiquitinated p53 is more abundant in RNF43 transfectants as compared with the control which suggests that RNF43 promotes p53 ubiquitination and degradation. Moreover, an increase in p53 ubiquitination was observed when RNF43-Flag-HA was co-expressed with NP-GFP as opposed to a diminished level of ubiquitinated p53 in only NP-GFP-transfected cells. This convincingly advocates the role of RNF43 in p53 regulation which is targeted by NP to stabilize p53 in IAV-infected cells.

### RNF43 mitigates NP-driven enhancement of p53 transcriptional activity

**Figure 6:**
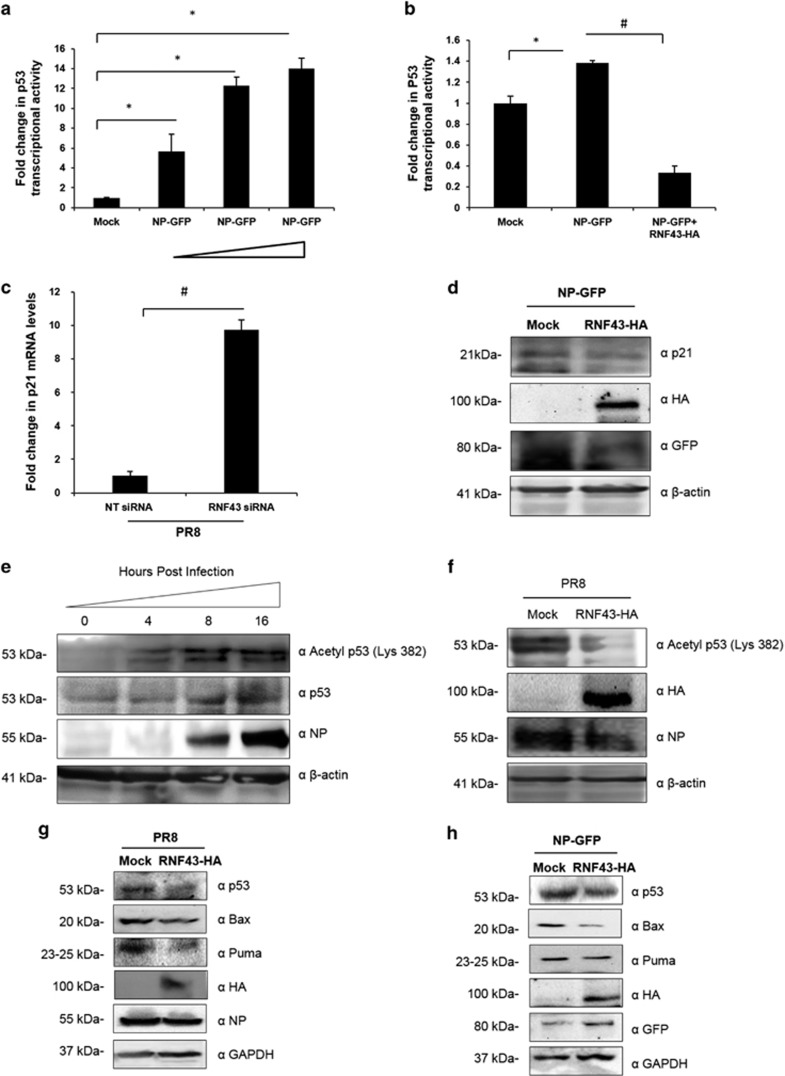
RNF43 decreases NP driven increased p53 transcriptional activity and signaling in the cells. (**a**) A549 cells were transfected with p21-Luc reporter plasmid, with or without growing amounts of pEGFP-NP (500 and 750 ng, and 1 μg) in conjunction with pcDNA-p53 and a control plasmid, Renilla luciferase pRL-TK. Luciferase activity of cell lysates of transfectants was analyzed. (**b**) A549 cells were transfected with p21-Luc reporter plasmid, pcDNA-p53 and a control plasmid, Renilla luciferase pRL-TK along with plasmids pEGFPN1 (mock), pEGFP-NP (NP-GFP) and pCDNA3.1-RNF43-Flag-HA (RNF43-HA) in the indicated combinations. Luciferase activity was measured. (**c**) HEK293T cells were treated with NT siRNA or RNF43 siRNA for 24 h followed by PR8 IAV infection at 1 MOI. Cells were harvested at 24-h post infection and total RNA was extracted followed by p21 mRNA estimation with qRT-PCR. (**d**) A549 cells were transiently transfected with pEGFP-NP with or without pCDNA3.1-RNF43-Flag-HA and were harvested at 48 h followed by SDS-PAGE. Western blotting was done using indicated antibodies. (**e**) A549 cells were seeded in a 6-well plate and were infected with PR8 virus at 1 MOI. Cells were harvested at 0, 4, 8, 16 and 24-h post infection, and subjected to Western blotting with indicated antibodies. (**f**) A549 cells were transfected with pCDNA3.1 (mock) and pCDNA3.1-RNF43-Flag-HA (RNF43-HA) plasmids followed by PR8 infection, 24-h post transfection. The cells were harvested after 24 h followed by Western blot analysis with anti-acetyl p53, HA, NP and *β*-actin antibodies. (**g**) A549 cells were transiently transfected with pCDNA3.1 (mock) or pCDNA3.1-RNF43-Flag-HA (RNF43-HA) plasmid constructs and after 24 h of incubation, cells were infected with PR8 virus at an MOI of 1 for 24 h. (**h**) Similarly, A549 cells were transiently transfected with pEGFP-NP with or without pCDNA3.1-RNF43-Flag-HA and were incubated for 48 h. (**g** and **h**) Cells were harvested and processed for Western blot analysis with indicated antibodies. Results in **a**–**c** are shown as mean±S.D. three independent experiments. * and ^#^ indicate statistically significant difference at *P*<0.05 and *P*<0.01, respectively

Stabilization of p53 by NP prompted us to look into the probable role of NP/RNF43 interaction in induction of p53-mediated cell functions. Tumor suppressor protein p53 is a transcription factor that regulates the expression of many genes that are crucial in mediating its tumor suppressing activity.^20, 21^ Therefore, we checked a dose-dependent effect of NP on p21 transcription which is under a direct control of p53 transcriptional activity.^33^ A549 cells were transiently co-transfected with p21 luciferase reporter plasmid containing the p53 binding site on its promoter together with indicated concentrations of pEGFP-NP and pcDNA-p53 and a control plasmid, Renilla luciferase pRL-TK. The p21 luciferase activity was measured and normalized to Renilla luciferase activity. p21 is a direct target gene of p53 hence a dose-dependent increase in p21 luciferase activity as compared with the mock transfectant suggests an increased p53 transcriptional activity (Figure 6a). To probe the significance of NP/RNF43 interaction in NP-driven enhanced transcriptional activity of p53, using the similar approach, A549 cells were co-transfected with pEGFPN1 or pEGFP-NP expression plasmid alone or together with RNF43-HA-expressing plasmid. As speculated, co-expression of RNF43 with NP resulted in an ~0.2-fold decrease in p53 transcriptional activity as compared with only NP-expressing cells (Figure 6b).

We checked the mRNA levels of p21 in RNF43 knocked down HEK293T cells infected with PR8. As compared with NT siRNA-treated cells, p21 mRNA levels were almost 10-fold higher in RNF43 depleted cells (Figure 6c). p21 protein levels were also observed to be undergoing a notable decrease in the presence of RNF43 when co-expressed along with NP-GFP (Figure 6d).

Acetylation of p53 is an important step to enable p53-mediated transactivation of different factors towards cellular functions.^34, 35^ We monitored the acetylation of p53 (Lys-382) at different time points post PR8 infection in A549 cells. Acetylation levels of p53 were observed to undergo a time-dependent change as shown in Figure 6e. We further assessed the effect of RNF43 on p53 acetylation in IAV-infected cells. To achieve the same, we transfected A549 cells with pCDNA3.1 (mock) and pCDNA3.1-RNF43-Flag-HA (RNF43-HA) plasmids followed by PR8 infection after 24 h. Cells were harvested after 24 h of infection followed by Western blot analysis. A decrease in p53 acetylation in the cells ectopically expressing RNF43 (Figure 6f) is likely to be the result of the concomitant increase in RNF43-mediated p53 ubiquitination.

Furthermore, A549 cells were infected with PR8 virus after being transfected with or without RNF43-Flag-HA-expressing plasmid construct. 24 h.p.i intracellular protein levels of p53 and its regulated molecules, Bax and Puma were inspected through Western blot analysis. In RNF43-expressing cells, there was an evident decrease in the protein levels of p53 and its downstream effectors Bax and Puma (Figure 6g). The same outline was observed when pEGFP-NP was transfected in conjunction with pCDNA3.1 or pCDNA3.1-RNF43-Flag-HA plasmids (Figure 6h).

p21, Bax and Puma are p53-regulated mediators of the latter's cellular functions.^20, 21^ These findings confirm the role of NP/RNF43 interaction in activation of p53 signaling pathways.

### RNF43 attenuates IAV NP-induced cell death

**Figure 7:**
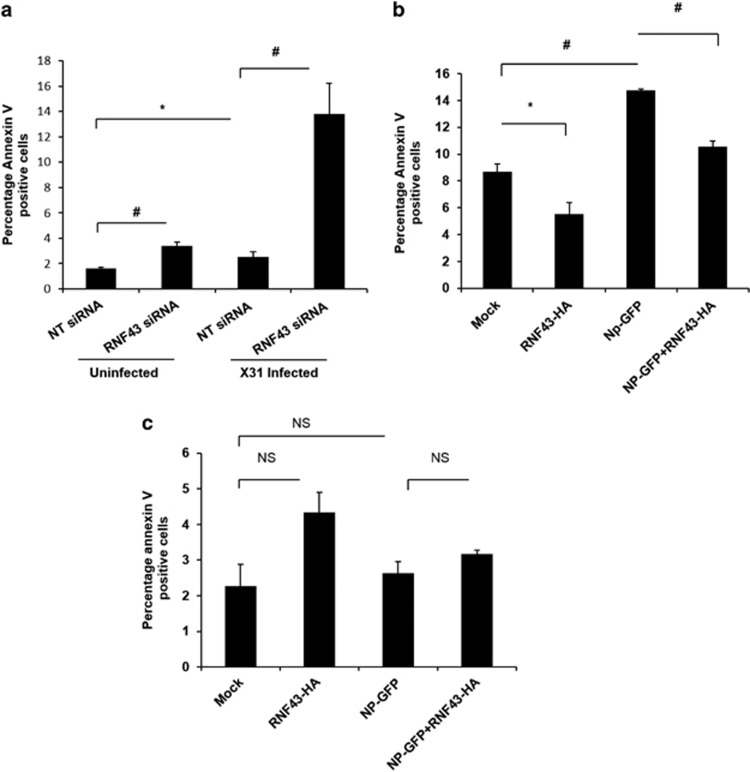
RNF43 attenuates IAV NP-induced cell death. (**a**) A549 cells were treated with NT siRNA or RNF43 siRNA for 24 h followed by X-31 IAV infection at 1 MOI. Cells were harvested at 24 h.p.i, stained with Annexin V FITC and subjected to flow cytometry. The percentage of Annexin V positive population is plotted on the graph. (**b**) A549 cells were transiently transfected with the indicated combinations of plasmids and 48 h post-transfection cells were harvested and processed for flow cytometric analysis of Annexin V FITC stained population which is plotted on the graph. (**c**) Same experiment was conducted in p53^−/−^ HCT116 cells. All graphs represent mean±S.D. of three independent experiments. * and ^#^ indicate statistically significant difference at *P*<0.05 and *P*<0.01, respectively, whereas NS refers to non-significant difference

To probe the effect of RNF43 on the induction of apoptosis in IAV-infected cells, flow cytometry-based Annexin V FITC labeling was performed. A549 cells were treated with NT siRNA or RNF43 siRNA and after 24 h cells were infected with IAV and subjected to annexin V staining. A remarkable 13% of total cell population showed annexin V staining in RNF43 siRNA-treated IAV-infected cells as against the 2.5% annexin V positive population in NT siRNA-treated IAV-infected cells. Thus, confirming the anti-apoptotic role of RNF43 in IAV-infected cells (Figure 7a).

The role of NP/RNF43 interaction in cell death induced by NP was further elucidated in NP microenvironment. Fourteen percent of pEGFP-NP transfectants displayed annexin V staining whereas RNF43 overexpression along with NP lowered the annexin V population to 10% (Figure 7b).

To investigate the exclusive role of p53 in NP/RNF43 interaction governed apoptosis, p53 null human colon cancer cells, HCT116 (p53^−/−^ HCT116) were transfected with pCDNA3.1 or pCDNA3.1-RNF43-Flag-HA with or without pEGFPN1 or pEGFP-NP. Annexin V staining of pEGFP-NP transfectants showed no significant difference as compared with the vector control. Similarly, RNF43 co-expression did not cause any change in the annexin V staining profile of NP-GFP-expressing cells (Figure 7c). This highlights the critical importance of p53 for carrying out NP-induced apoptosis. Most importantly, it draws attention to the critical role of RNF43 in virus-induced p53-mediated apoptosis.

## Discussion

Ubiquitination is a key regulatory mechanism in the orchestration of various cellular activities including signal transduction, transcription, membrane protein trafficking, apoptosis, autophagy and immune responses.^36^ Hence, it has become increasingly evident that the comprehensive ubiquitinylation process remains a prime target of a broad range of pathogens including viruses. Viruses may encode E3 ligases or deubiquitinases or may redirect the cellular E3s and deubiquitinases to modulate the ubiquitination status of substrates of choice.^11^

The present study for the first time provides evidence that NP interacts with RNF43, a member of RING finger family of cellular E3 ligases that mark proteins for ubiquitination. This interaction was validated in transfected mammalian cells and was also found to be conserved between PR8 and X-31 IAV strains. The conserved nature of the NP–RNF43 interaction underlines its significance in viral replication and pathogenesis.

We also report that IAV NP and RNF43 predominantly co-localize in the nucleus. IAV infection attenuated RNF43 mRNA as well as protein expression and overexpression of RNF43 counteracts IAV NP leading to a dose-dependent decrease in NP mRNA and vRNA. There was a drastic increase in viral replication under the influence of RNF43 knockdown. The decline in RNF43 mRNA and protein levels on IAV infection correlates with the decrease in viral replication by RNF43. It substantiates its role as an antiviral host protein. E3 ligases, highly specific mediators of ubiquitination, are extensively exploited by viruses to carry out replication and pathogenesis. It is of utmost importance to this study that many other viruses including human oncogenic papillomavirus, HIV and simian virus 40 also either target host ubiquitin ligases or encode ubiquitin ligases to divert their pathway of regulation by altering their specificity.^11, 37, 38^ Influenza virus too exploits the host ubiquitination status of many cellular factors to evade host defense mechanisms and achieve maximum replication efficiency.^12, 13^ Thus, it is concluded that RNF43 is targeted by the virus for a strategic attenuation of RNF43 for efficient viral replication and pathogenesis.

Wang *et al.* illustrated the stabilization of p53 by NP through compromised MDM2-mediated ubiquitination.^14^ In our study, we found that NP-driven stabilization of p53 was impeded by RNF43 via a decreased p53 half-life and a noteworthy augmentation of the ubiquitinated p53 levels in the RNF43 microenvironment. This can be attributed to the post-translational regulation of p53 by RNF43 through targeting p53 for ubiquitination. These results are in coherence with the fact that RNF43 interacts with p53^19^ and the siRNA caused downregulation of RNF43 led to a significant increase in p53 protein expression in HepG2 and SMMC-7721 cells.^16^ Several E3 ligases along with MDM2 are known to regulate p53 ubiquitination, for example, Pirh2, MdmX, HAUSP, ARF, COP1 and ARF-BP1.^22, 23^ Thus, the current study unravels a novel mechanism of p53 regulation via RNF43. Although, a separate study can be undertaken to explore RNF43-mediated p53 regulation but our study provides compelling evidences that RNF43 targets p53 for ubiquitination.

**Figure 8:**
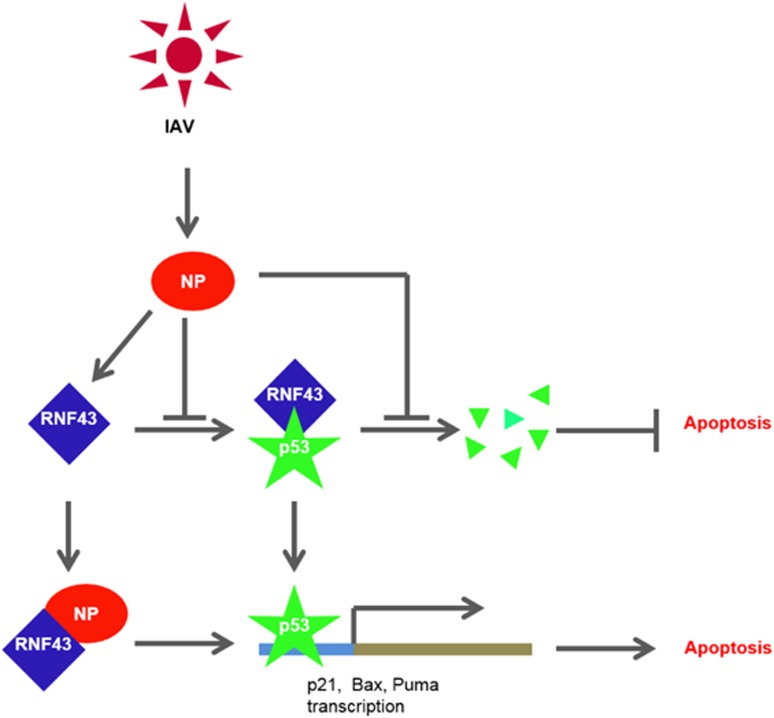
The proposed model for the role of NP/RNF43 interaction in regulation of p53-mediated cell death by IAV NP. The proposed model for the regulation of p53-mediated cell death by IAV NP. p53 is proposed to be regulated by RNF43 through ubiquitylation resulting in its destabilization. NP interacts with RNF43 thereby preventing ubiquitination of p53 and causing its stabilization and accumulation inside the infected cell resulting into activation of p53 signaling cascade including p21, Bax, Puma and eventually results in cell death in IAV-infected cell

Apoptosis induction is one of the hallmarks of IAV infection. It is considered to be host's defense against the virus but at the same time it is hypothesized that the appropriately timed apoptosis controlled by IAV is important for efficient viral replication.^39^ Accumulation of p53, a critical mediator of apoptosis, in IAV-infected cells is a well-documented phenomenon and in fact is reported to accumulate in a biphasic pattern.^40^ Our study also demonstrates an accumulation of p53 in IAV-infected cells along with an increasing acetylation levels that corresponds to an increased p53 activity. Turpin *et al.*^41^ demonstrated that p53 is required for IAV-induced cell death but at the same time was important in IFN induction. However, IFN-dependent antiviral response of p53 is reported to be independent of its pro-apoptotic functions.^26^ In the midst of these conflicting notions regarding the role of p53 in IAV replication and pathogenesis, our study eloquently proves the critical importance of p53 in NP-mediated apoptosis. We previously reported the role of NP in inducing host cell death.^42^ Stabilization and accumulation of p53 by NP can be accredited to the pro-apoptotic nature of NP, which, in this case hijacks RNF43 and withholds its regulatory effects on p53. Together with these findings, the data described here points to a model in which IAV increases p53 downstream signaling and apoptosis by suppressing the RNF43-mediated ubiquitination of p53 (Figure 8). Hence, interaction of NP with RNF43 to modulate p53 ubiquitination is a proof of direct mechanism of apoptosis induction by IAV.

Furthermore, the enhanced p21 transcription under the effect of NP suggests that NP could be involved in carrying out cell cycle arrest following IAV infection^43^ thus, paving the way for exploring the role of NP in causing IAV-driven cell cycle arrest.^44, 45^ Lastly, owing to its antiviral properties and the conserved nature of IAV NP–RNF43 interaction, RNF43 could indeed prove to be an attractive antiviral developing target.

## Materials and Methods

### Cell culture and viruses

All the cell lines were grown and maintained in Dulbecco's modified Eagle's medium (DMEM; Hyclone, Logan, UT, USA) supplemented with 10% fetal calf serum (Hyclone), penicillin–streptomycin solution (100 units per ml; Invitrogen, Grand Island, NY, USA) in 5% CO_2_-containing environment. A/*Puerto Rico*/8/34 (PR8) and A/*Aichi*/2/1968 (X-31) strains of IAV were used for viral infection at an MOI of 1 unless specified otherwise.

### Transfection and IAV infection

DNA transfections were done with Lipofectamine 2000 (Invitrogen) in NANS medium and after 6 h of incubation; NANS was replaced with 10% fetal calf serum containing DMEM. For virus infections, DMEM supplemented with 0.3% bovine serum albumin (BSA; GIBCO, Grand Island, NY, USA) was used. Cells were washed with phosphate-buffered saline (PBS) followed by virus infection and incubated for virus adsorption at 37 °C. One hour later the virus was removed and cells were incubated with DMEM-containing 0.3% BSA. In mock-infected cells, virus was replaced with PBS.

### Plasmids, siRNA and antibodies

NP gene from A/Chicken/Hatay/2004 (H5N1) influenza virus was cloned into pEGFPN1 vector and also in pCDNA3.1-myc vector. Full-length RNF43, cloned into pCDNA3.1-Flag-HA was provided by Clevers and coworkers.^17^ RNF43 was also cloned into pYESTrp2 for yeast two-hybrid assay. p53-GFP, MDM2-Flag and Ub-His were kind gifts from Ma and coworkers.^14^ p21 luciferase reporter plasmid was provided by Vogelstein and coworkers.^33^ Pool of gene specific siRNA against RNF43 was purchased from Santa Cruz, CA, USA. Anti-RNF43 (sc-165398), anti-M2, anti-p53, anti-p21, anti-HA, anti-GAPDH and anti-GFP antibodies were purchased from Santa Cruz Biotechnology, CA, USA. Anti-RNF43 (SAB2102033) and anti-*β*-actin antibodies were purchased from Sigma-Aldrich (St. Louis, MO, USA). Anti-Bax and anti-Puma antibodies were purchased from Cell Signaling (Boston, MA, USA). Anti-acetyl p53 (Lys-382) was purchased from Biolegend, San Diego, CA, USA.

### Yeast two-hybrid screening

The bait plasmid was constructed by cloning IAV NP coding sequence in-frame with the LexA DNA-binding domain in pHybLexA/Zeo. The prey plasmid was prepared by cloning RNF43 into pYESTrp2. Yeast two-hybrid screening was conducted as described previously.^4^

### Western blot analysis

Cells were harvested in lysis buffer (50 mM Tris, pH 7.5, 150 mM NaCl, 1 mM EDTA and 0.1% NP-40) supplemented with complete protease inhibitor cocktail (Roche Diagnostics, Pleasonton, CA, USA). The lysates obtained were then subjected to SDS-polyacrylamide gel electrophoresis (PAGE).

### Co-immunoprecipitation

Cells harvested in the above mentioned lysis buffer were incubated with the primary antibody overnight at 4 °C which was followed by an incubation of 90 min with protein A or protein G sepharose beads (GE Healthcare, Uppsala, Sweden). Beads were then washed three times with chilled PBS. Immunoprecipitates were eluted through boiling the beads in SDS-PAGE sample buffer for 10 min, subjected to SDS-PAGE followed by western blot analysis.

### Immunofluorescence microscopy

Cells infected with IAV at 5 MOI or transfected with expression plasmid pEGFP-NP, were fixed in 4% paraformaldehyde for 15 min and permeabilized with 0.4% Triton X-100 in PBS for 20 min at room temperature. Fixed cells were then washed twice with PBST (0.2% Tween 20), blocked with 2% BSA for 1 h at room temperature and left at 4 °C for overnight immunostaining with specific primary antibody in 0.5% BSA. Unbound antibodies were washed away with PBST and incubated with secondary Alexa Fluor antibodies (594 488; Invitrogen) solution in 0.5% BSA for 1 h and washed with PBST. Slides were prepared by mounting the cover slips with Prolong Gold anti-fade medium with nuclei staining dye, DAPI (Invitrogen) and sealing the sides of the cover slips. Cell images were taken under × 60 objective lens of Leica DM6000B confocal microscope. Images were processed using NIS Elements AR 3.0 software (Nikon, Melville, NY, USA).

### Quantitative real-time PCR analysis

Total RNA was isolated from cells using TRIzol reagent as per the supplier's instructions (Invitrogen). Reverse transcription of 5 μg of mRNA was performed with M-MuLV reverse transcriptase (Fermentas, Waltham, MA, USA) in a 50 μl volume reaction, according to the manufacturer's guidelines. cDNA obtained was diluted 1 : 10 and 2 μl was used in Syber green based quantitative real-time PCR reaction in a volume of 20 μl using the instrument StepOnePlus Real-Time PCR System (Applied Biosystems, Foster City, CA, USA). Primers used for real-time RT-PCR are RNF43 primers forward GCAGGAGCTACGGGTCATTTC and reverse GATGCTGATGTAACCAGGGGT; NP vRNA primer, CTC GTC GCT TAT GAC AAA GAA G, and NP gene primers (for mRNA) forward CTC GTC GCT TAT GAC AAA GAA G and reverse AGA TCA TCA TGT GAG TCA GAC; p21 primers, forward GTCAGTTCCTTGTGGAGCCG and reverse CTCCAGTGGTGTCTCGGTG; p53 primers, forward GTTTCCGTCTGGGCTTCTTGC and reverse ACGCAAATTTCCTTCCACTCGG; housekeeping gene, GAPDH primers, forward TCA CTG CCA CCC AGA AGA CTG and reverse GGA TGA CCT TGC CCA CAG C. All qRT-PCRs were performed in triplicates. Fold change in mRNA levels were calculated using the delta–delta threshold cycle (ΔΔCT) method after normalizing with GAPDH.

### Luciferase reporter assay

Luciferase activity was measured using the Dual Luciferase Reporter Assay System (Promega) according to the manufacturer's instructions (Promega). The firefly luciferase activity of each sample was normalized to constitutively expressed Renilla luciferase plasmid, pRL-TK. Similarly, for *Renilla* luciferase activity, firefly luciferase plasmid pGL3 was used as an internal control.

### Cycloheximide assay

Cycloheximide (Sigma-Aldrich), a protein translation inhibitor, was added to the media at a concentration of 50 *μ*g/ml, 44-h post transfection in A549 cells. Cells were incubated for indicated time periods at 37 °C and harvested in SDS-PAGE sample buffer and subjected to SDS-PAGE followed by western blot analysis.

### Ubiquitination assay and purification of His-tagged ubiquitin conjugates

Proteasome inhibitor, Mg132 was used to determine the ubiquitination status of p53 protein. HEK293T cells grown in 60 mm cell culture dishes were transiently co-transfected with the indicated plasmids and treated with 20 μM MG132 (Calbiochem, EMD Biosciences, Billerica, MA, USA) for 5 h before harvesting. Cells were lysed in Triton X-100 buffer (50 mM Tris, pH 7.5, 150 mM NaCl, 1 mM EDTA and 0.1% Triton X-100) and mixed with SDS-PAGE sample buffer followed by western blot analysis with anti-p53 antibody. For purification of His-tagged conjugates, HEK293T cells were treated similarly and were processed as described previously.^46^ Concisely, the cells were lysed in a lysis buffer (6 M guanidinium–HCl, 0.1 M Na_2_HPO_4_–NaH_2_PO_4_, 0.01 M Tris-HCl, pH 8.0, 5 mM imidazole, 10 mM *β*-mercaptoethanol) followed by an incubation of 12 h with Ni^2+^–NTA beads (Qiagen, Hilden, Germany) at 4 °C on a rotating wheel. The beads were then washed as explained previously and incubated with an elution buffer (200 mM imidazole, 0.15 M Tris-HCl, pH 6.7, 30% glycerol, 0.72 M *β*-mercaptoethanol and 5% SDS) for 20 min at room temperature. The eluates were mixed with SDS-PAGE sample buffer and analyzed with western blotting with required antibodies.

### Flow cytometry

Annexin V staining of cells was done with Annexin V FITC Assay kit (Cayman Chemicals, Ann Arbor, MI, USA), according to the manufacturer's instructions. Samples were acquired on BD FACS Calibur Flow cytometer (BD Biosciences, Franklin Lakes, NJ, USA) and analyzed using Flowjo version 9.3.3 software (Tree Star Inc., Ashland, OR, USA).

### Statistical analysis

Data are expressed as mean±S.E. Means were compared by one-factor analysis of variance followed by Fisher protected least significant difference to assess specific group differences. Differences were considered significant at *P*<0.05.

## Abbreviations

IAV: influenza A virus
NP: nucleoprotein
RNF43: RING Finger 43
Ub: ubiquitin
UPP: ubiquitin proteasomal pathway
