# Supplementary material for: The nucleoprotein of influenza A virus induces p53 signaling and apoptosis via attenuation of host ubiquitin ligase RNF43
Source: Cell Death Dis. 2015 May 21;6(5):e1768–. doi: 10.1038/cddis.2015.131 (PMC4669709; doi:10.1038/cddis.2015.131)
Supplement: Supplementary Figure 1 [file cddis2015131x1.pdf]

Supplementary Figure S1.

(A)

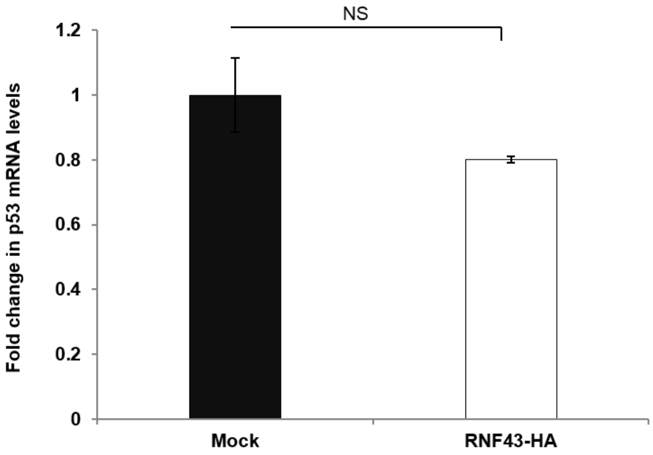

Fig. S1. RNF43 does not alter p53 transcripts levels. *A549 cells were transfected with plasmids pCDNA3.1 (Mock) or pCDNA3.1-RNF43-Flag-HA (RNF43-HA). Cells were harvested after 48h post transfection and processed for mRNA quantification by qRT PCR analysis. Result is shown as mean ± SD of three independent experiments. NS indicates non-significant statistical difference.*
